# Distinct Roles of Transketolase (TktA) and Transaldolase (talB) in Metabolism, Biofilm Formation, and Flea Colonization in *Yersinia pestis*

**DOI:** 10.3390/pathogens15060603

**Published:** 2026-06-03

**Authors:** Amélie Dewitte, Maurane Dégardin, Ivan Nemazanyy, Florent Sebbane, Sébastien Bontemps-Gallo

**Affiliations:** 1 Univ. Lille, CNRS, Inserm, CHU Lille, Institut Pasteur de Lille, U1019-UMR 9017-CIIL-Center for Infection and Immunity of Lille, 59000 Lille, France; 2 Platform for Metabolic Analyses, Structure Fédérative de Recherche Necker, INSERM US24/CNRS UAR 3633, 75015 Paris, France

**Keywords:** *Yersinia pestis*, flea vector, pentose phosphate pathway, transketolase (TktA), transaldolase (TalB), metabolomics, biofilm formation, metabolic adaptation

## Abstract

The flea-borne transmission of *Yersinia pestis* relies on biofilm formation and metabolic adaptation within the insect gut. The pentose phosphate pathway (PPP) is central to these processes, yet the contribution of its non-oxidative branch remains poorly defined. Here, we investigated the roles of transketolase (TktA) and transaldolase (TalB) in plague bacillus physiology, metabolism, and flea colonization. TktA was essential for growth, preventing assessment of its role in biofilm formation and in vivo colonization. In contrast, TalB was dispensable for growth but required for optimal biofilm formation. In fleas, the Δ*talB* mutant colonized the proventriculus but displayed a lower bacterial load than the wild-type strain at later time points, indicating a defect in sustained colonization. Metabolomic analyses revealed that disruption of *tktA* severely impairs PPP-associated metabolism, whereas loss of *talB* is associated with disruption of nucleotide homeostasis, carbon redistribution toward glycolysis, and a redox imbalance. These findings demonstrate a functional partitioning of the non-oxidative PPP and identify it as a key metabolic control node linking metabolism to colonization dynamics in *Y. pestis*.

## 1. Introduction

*Yersinia pestis*, the etiological agent of plague, alternates between mammalian hosts and flea vectors, where it undergoes profound physiological and metabolic adaptations [1,2,3]. Following a blood meal taken by the insect from a bacteremic host, the plague bacillus passes successively through the esophagus and the proventriculus—a valve that rhythmically contracts—before reaching the midgut [3,4,5,6]. Within a few hours, a transient bactericidal soft mass forms in the proventriculus and entraps the bacteria [7]. During subsequent blood meals, this mass is partially dislodged; however, residual bacteria promote the formation of a new mass that is progressively consolidated through the production of bacterial extracellular polysaccharides [6,7,8,9]. This structure eventually becomes sufficiently solid to obstruct the passage of blood into the midgut, leading to the so-called “blocked flea” phenotype [4,6,7]. During subsequent feeding attempts, ingested blood cannot be absorbed into the midgut, becomes contaminated upon contact with the bacterial mass, and is regurgitated at the bite site, thereby enabling transmission to a new host [4].

The establishment of a transmissible infection relies on the ability of *Y. pestis* to sustain growth within the insect while producing poly-β-1,6-N-acetyl-D-glucosamine, a key component of the biofilm matrix [3,6,10]. *Y. pestis* benefits from a highly specific nutritional environment generated by partial digestion of the blood meal [11]. Several studies have highlighted the importance of bacterial metabolism and its regulation in flea colonization [12,13,14,15,16]. In particular, microarray data indicate that *Y. pestis* undergoes extensive metabolic reprogramming in the flea gut, shifting from hexose utilization to the catabolism of amino acids (including proline, histidine, glutamine, arginine, and spermidine) [14]; however, direct experimental evidence for this metabolic shift in *Y. pestis* within the flea is lacking.

**Figure 1:**
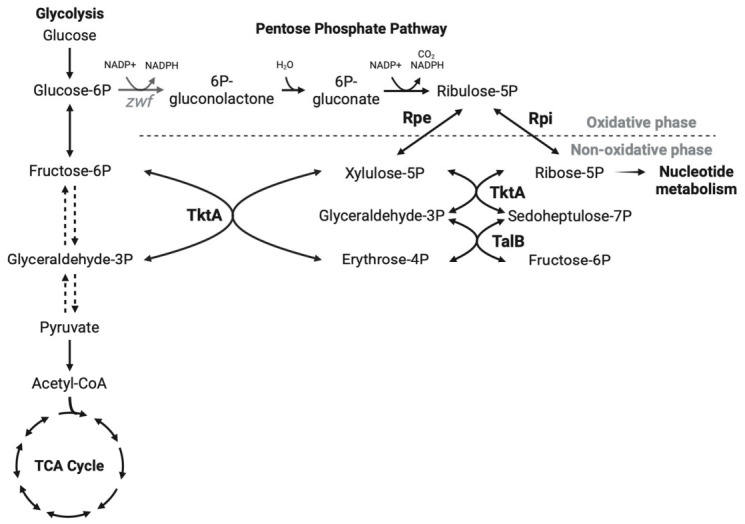
Integration of glycolysis, pentose phosphate pathway, and nucleotide metabolism in Enterobacteriaceae. The schematic illustrates the metabolic connections between glycolysis, the pentose phosphate pathway (PPP), and the tricarboxylic acid (TCA) cycle in Enterobacteriaceae. Glucose-6-phosphate can be directed toward glycolysis or enter the oxidative phase of the PPP via Zwf, generating NADPH and producing ribulose-5-phosphate. However, in *Y. pestis*, Zwf has been proposed to be non-functional due to a missense mutation, potentially impairing oxidative PPP flux [17]. The non-oxidative phase interconverts pentose phosphates through the action of Rpe and Rpi, generating ribose-5-phosphate for nucleotide biosynthesis. Carbon rearrangements mediated by transketolase (TktA) and transaldolase (TalB) connect PPP intermediates (xylulose-5-phosphate, erythrose-4-phosphate, and sedoheptulose-7-phosphate) to glycolytic intermediates (fructose-6-phosphate and glyceraldehyde-3-phosphate). These reactions enable bidirectional carbon flux between pathways, supporting redox homeostasis, central carbon metabolism, and nucleotide synthesis. Dashed arrows indicate indirect or multistep connections. Created in BioRender.

The pentose phosphate pathway (PPP) is a central metabolic network that connects carbon flux, nucleotide biosynthesis, and redox homeostasis (Figure 1). In *Y. pestis*, the PPP has been implicated in flea colonization, notably through the role of ribose-5-phosphate isomerases (RpiA) in biofilm formation and transmission [7]. However, the PPP is not a uniform pathway. While its oxidative branch primarily generates NADPH, the non-oxidative branch redistributes carbon intermediates through reversible sugar-phosphate interconversions linking glycolysis to biosynthetic pathways. Whether this non-oxidative branch functions solely as a source of metabolic intermediates or instead acts as a regulatory hub coordinating bacterial adaptation during flea colonization remains unknown.

Two enzymes, transketolase (TktA) and transaldolase (TalB), catalyze key carbon rearrangements within the non-oxidative PPP and thereby connect central carbon metabolism to nucleotide biosynthesis and redox balance. Despite their central biochemical roles, their contribution to bacterial adaptation in the flea has not been fully investigated. Notably, previous work has suggested that the Δ*talB* mutant plays a role in processes underlying transmission [7].

Here, we investigated the respective roles of TktA and TalB in *Y. pestis* physiology and flea colonization. We show that these enzymes play distinct and non-redundant roles: TktA acts as an essential metabolic hub required for bacterial growth, whereas TalB is dispensable in vitro but contributes to biofilm formation in vitro and sustained colonization in the flea. Through combined metabolomic and infection analyses, we show that TalB supports metabolic homeostasis by coordinating nucleotide balance, carbon flux distribution, and redox state. Our findings reveal that the non-oxidative PPP is not merely a biosynthetic pathway but a central metabolic control node that governs bacterial adaptation, linking core metabolism to colonization dynamics and transmission within the flea vector.

## 2. Materials and Methods

### 2.1. Strains, Mutants, Plasmids, and Growth Conditions

The bacterial strains and plasmids used in this study are listed in Table 1. Mutant strains were complemented by electroporation with the high-copy cloning plasmid pCR-Blunt harboring either *tktA* or *talB*.

Strains were grown in lysogeny broth (LB; 240230, BD), brain heart infusion (BHI; 211059, BD) or M9 (M9 Minimal salts, 5×, M9956, Merck, supplemented with 1 mM thiamine hydrochloride, 0.2% casamino acids, 2 mM MgSO_4_, 0.1 mM CaCl_2_) at 21 °C. When required, agar (15 g·L^−1^), kanamycin (50 µg·mL^−1^), zeocin (50 µg·mL^−1^), or ampicillin (100 µg·mL^−1^) were added to obtain solid and/or selective media.

To compare growth rates, bacteria were cultured at 21 °C, and growth in BHI or M9 was monitored by measuring the optical density at 600 nm (OD600) using a Density Meter Ultraspec 10 Cell (Biochrom, Cambridge, UK).

### 2.2. Assessment of Biofilm Formation In Vitro

Bacteria were incubated in BHI for 28 h at 21 °C with shaking at 180 rpm in 24-well plates (1 × 10^7^ bacteria in 1 mL). Following incubation, the medium was removed, and adherent biofilms were stained with 0.01% crystal violet for 15 min at room temperature. Wells were then gently rinsed three times with distilled water (ddH_2_O) to remove non-adherent cells. The retained dye was solubilized using an ethanol–acetone solution (80:20, *v*/*v*). The resulting solution was transferred to a 96-well plate, and absorbance was measured at 542 nm using a Victor X3 plate reader (PerkinElmer, Shelton, CT, USA).

### 2.3. Flea Infection, Co-Infection Assays, and Proventriculus Analysis

Flea infection was performed as previously described in a method chapter [9,19]. Starved *Xenopsylla cheopis* fleas were allowed to feed on heparinized mouse blood containing 5 × 10^8^ bacteria per mL (grown at 37 °C overnight in BHI), using an artificial apparatus (feeder) for 1 h. To evaluate proventriculus colonization, female fleas were infected with *Y. pestis* expressing green fluorescent protein (GFP) from the pAcGFP plasmid (Addgene, Cambridge, MA, USA). When required, 19 to 21 fleas were randomly selected and dissected under a stereomicroscope to isolate the digestive tract. Immediately after dissection, fluorescence images were acquired using an Eclipse Ci-S fluorescence microscope (Nikon, Tokyo, Japan) equipped with a DS-Fi1 camera (Nikon). Images were processed using ImageJ version 1.54g [9,19] and a macro allowing to determine the number of proventriculus infected, the surface colonized by the bacteria and the fluorescence intensity.

### 2.4. Metabolomic Analysis

Metabolite profiling of *Y. pestis* was performed by liquid chromatography-mass spectrometry (LC-MS) as previously described [20]. Bacteria were grown in BHI medium, and metabolic activity was rapidly quenched by immersion in liquid nitrogen for 10 s. For each condition, approximately 40 mg of bacterial dry weight was used for metabolite extraction. Metabolites were extracted using a cold solvent mixture of methanol/acetonitrile/water (50:30:20, *v*/*v*/*v*) at −20 °C. The volume of extraction solvent was adjusted proportionally to the bacterial biomass (dry weight), ensuring consistent extraction efficiency across samples. Samples were vortexed for 5 min at 4 °C and centrifuged at 16,000× *g* for 15 min at 4 °C. Supernatants were collected for analysis. Chromatographic separation was performed using a SeQuant ZIC-pHILIC column (Millipore, Darmstadt, Germany). The mobile phases consisted of (i) 20 mM ammonium carbonate with 0.1% ammonium hydroxide (aqueous phase) and (ii) acetonitrile (organic phase). A linear gradient from 80% organic to 80% aqueous phase was applied over 15 min at a flow rate of 200 μL/min, with the column maintained at 48 °C. Mass spectrometry detection was carried out using a Q-Exactive Plus mass spectrometer (Thermo, Waltham, MA, USA) operating in electrospray ionization mode with polarity switching. Data were acquired over a mass range of 75–1000 m/z at a resolution of 35,000 (at 200 m/z). Lock masses ensured mass accuracy below 5 ppm. Metabolite identification and peak integration were performed using TraceFinder software (Thermo), based on exact mass and retention time. Metabolomic data were analyzed using MetaboAnalyst 6.0 [21]. Principal component analysis (PCA) and heatmap visualizations were generated using normalized metabolite peak areas. For heatmap representation, metabolite values were subjected to row-wise centering and scaling, allowing visualization of relative differences in abundance for each metabolite across samples independently of absolute signal intensity. Metabolites were clustered using hierarchical clustering based on Pearson distance and Ward linkage. The data are available in Table S1.

## 3. Results

### 3.1. TktA and TalB Have Distinct Impacts on Bacterial Physiology

**Figure 2:**
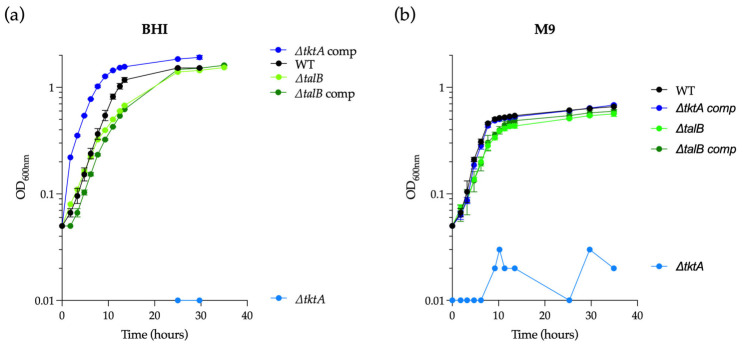
Bacterial growth in pentose phosphate pathway mutants. Growth curves of the wild-type (WT) strain and the indicated mutants in (**a**) rich media (BHI) and (**b**) define M9 broth. Data are expressed as optical density (OD_600nm_). Data points represent mean ± standard deviation from three independent biological replicates. Colors correspond to the different strains as indicated in the legend.

To assess the contribution of the non-oxidative PPP to bacterial physiology, we compared the growth of ∆*tktA* and ∆*talB* mutants in rich medium (BHI) and defined medium (M9) (Figure 2). The ∆*tktA* mutant failed to initiate growth in BHI or M9, indicating that transketolase is essential for bacterial replication under these conditions. Complementation of *tktA* fully restored growth, confirming that the observed defect was specifically due to the loss of TktA. In contrast, the ∆*talB* mutant exhibited growth comparable to that of the wild-type strain, with no major defect in growth kinetics. These results indicate that, unlike TktA, TalB is dispensable for bacterial growth in vitro. Together, these findings demonstrate that the non-oxidative branch of the pentose phosphate pathway is functionally partitioned, with TktA acting as an essential metabolic enzyme required for growth, whereas TalB plays a non-essential role under these conditions.

### 3.2. Both TktA and TalB Contribute to Biofilm Formation In Vitro

**Figure 3:**
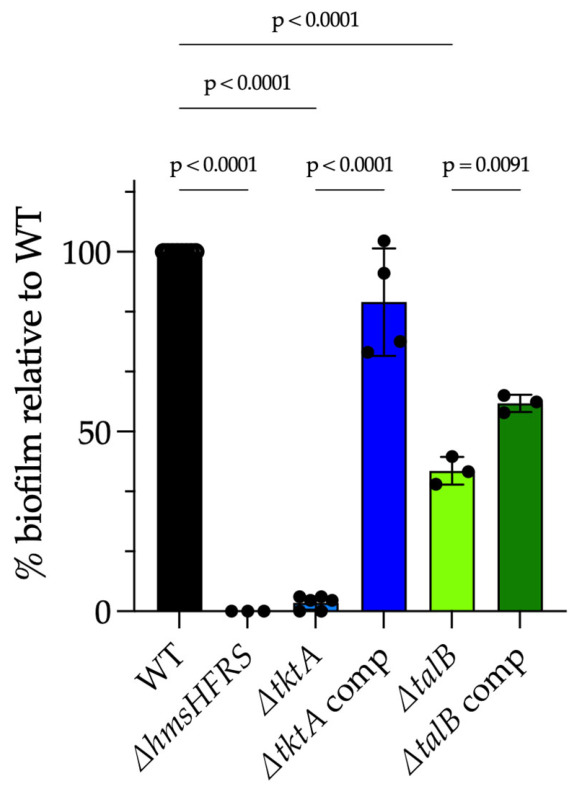
Biofilm formation by wild-type strain, ∆*tktA* and ∆*talB* mutant strains. Biofilm formation was quantified using a crystal violet assay following 28 h incubation in BHI at 21 °C. Bars represent the mean values from independent experiments, and dots indicate individual replicates. Error bars correspond to standard deviation. Statistical significance was assessed using One-way ANOVA, with *p* values indicating significance.

Given the central role of biofilm formation in flea blockage and transmission [3,6], we next evaluated the contribution of TktA and TalB using a crystal violet assay (Figure 3). The ∆*tktA* mutant did not produce detectable biofilm under these conditions. However, this phenotype is most likely a consequence of its inability to grow in vitro, precluding a direct assessment of its specific contribution in biofilm formation. Complementation of ∆*tktA* mutant restored biofilm formation to levels comparable to those of the wild-type strain. In contrast, the ∆*talB* mutant exhibited a significant but partial defect, forming approximately 60% less biofilm than the wild-type strain. Complementation of ∆*talB* mutant increased biofilm formation compared to the mutant, although levels remained lower than those of the wild-type strain. As this mutant displays normal growth in vitro, this reduction indicates a specific contribution of TalB to biofilm formation independent of growth defects. Overall, these data indicate a specific contribution of TalB to biofilm formation, whereas the role of TktA cannot be assessed independently of its growth defect under these conditions.

### 3.3. TalB Does Not Affect Initial Colonization but Differences Emerge During Later Stages of Proventriculus Colonization

**Figure 4:**
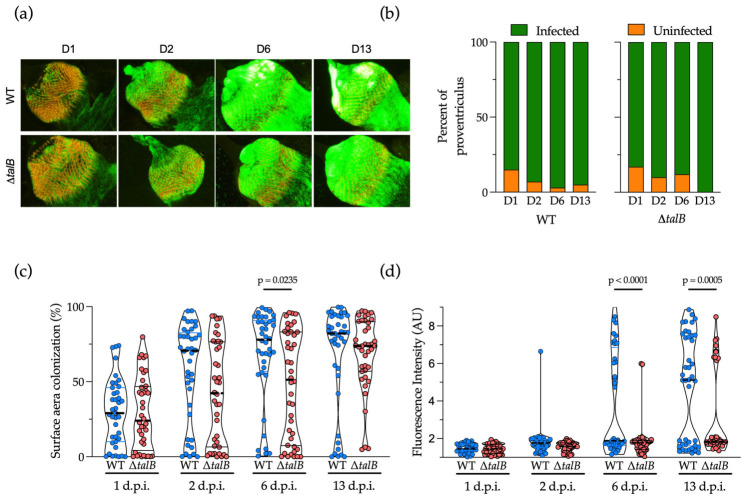
Colonization of the *X. cheopis* flea proventriculus by the wild-type strain and the ∆*talB* mutant strain. (**a**) Representative fluorescence images of the proventriculus (in orange) infected with the wild-type (WT) or the ∆*talB* mutant strain (in green) acquired at 1, 2, 6, and 13 days post-infection (B2-A filter, objective 20×). (**b**) The percentage of proventriculi colonized (in green) or not (in orange) by *Y. pestis.* (**c**) Changes over time in the surface area colonization (%) of the proventriculus and (**d**) the mean fluorescence intensity (arbitrary unit) in the proventriculus of fleas that fed on blood infected with the wild-type strain (in blue), the ∆*talB* mutant (in pink). Each dot corresponds to an individual flea (n = 19 to 21). Statistical significance was assessed using a Kruskal–Wallis test followed by Dunn’s multiple comparisons test and is indicated on the graphs.

Considering the blockage rate observed in the Δ*talB* mutant compared to the wild-type strain [7] and its role in biofilm formation in vitro (Figure 3), we next analyzed its ability to colonize the flea proventriculus using fluorescence microscopy and quantitative image analysis (Figure 4). The extent of colonization was first assessed by measuring the percentage of the proventricular surface covered by bacteria (Figure 4a). The proportion of infected proventriculi (i.e., containing detectable bacteria) was similar between the two strains over time (Figure 4b). At early and intermediate time points post-infection (days 1, 2, and 13), no significant difference in colonized surface area was observed between the Δ*talB* mutant and the wild-type strain (Figure 4c). A slight reduction was detected at day 6; however, overall, these results indicate that TalB is not required for the initial colonization of the proventriculus. In contrast, quantification of fluorescence intensity, used as a proxy for bacterial load, revealed a significantly lower signal in the Δ*talB* mutant at later time points (days 6 and 13) compared to the wild-type strain (Figure 4d). This reduction indicates that, although the mutant can successfully establish colonization, it is impaired in its ability to maintain or expand bacterial populations within the proventriculus over time. Together, these results indicate that TalB is dispensable for initial colonization but contributes to efficient colonization and maintenance of bacterial load within the flea foregut.

### 3.4. Disruption of the Non-Oxidative PPP Induces Distinct Metabolic Rewiring

**Figure 5:**
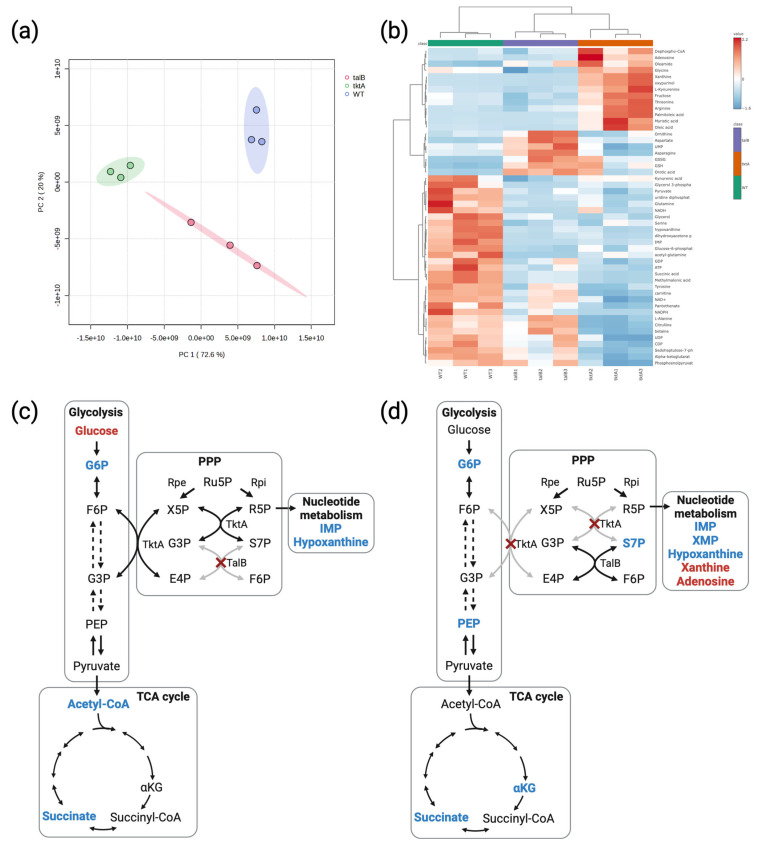
Metabolomic analyses of Δ*tktA* and Δ*talB* mutants compared to the wild-type strain. (**a**) Principal component analysis of metabolomic profiles from wild-type (WT, in purple), Δ*tktA* (in green), and Δ*talB* (in red) mutant strains. Each point represents an independent biological replicate. The percentage of variance explained by each principal component is indicated on the axes. (**b**) Heatmap representation of the top 50 most significantly altered metabolites across WT, Δ*tktA*, and Δ*talB* strains. Metabolite abundances were normalized by row-wise centering and scaling in MetaboAnalyst. Colors, therefore, represent relative differences in metabolite abundance for each metabolite across all samples, rather than absolute concentrations or direct fold changes relative to the wild-type strain (red, relatively increased abundance; blue, relatively decreased abundance). Metabolites were clustered using hierarchical clustering (Pearson distance, Ward linkage). (**c**) Schematic representation of central carbon metabolism in the Δ*talB* mutant. (red, increased relative to the wild-type; blue, decreased relative to the wild-type) (**d**) Schematic representation of metabolic alterations in the Δ*tktA* mutant (red, increased relative to the wild-type; blue, decreased relative to the wild-type). G6P (Glucose-6-phosphate); F6P (Fructose-6-phosphate); G3P (Glyceraldehyde-3-phosphate); PEP (Phosphoenolpyruvate); αKG (Alpha-ketoglutarate, 2-oxoglutarate); CoA (Coenzyme A); X5P (Xylulose-5-phosphate); E4P (Erythrose-4-phosphate); R5P (Ribose-5-phosphate); S7P (Sedoheptulose-7-phosphate); F6P (Fructose-6-phosphate); PC (Principal component). Created in BioRender.

Having established that inactivation of non-oxidative PPP enzymes differentially affects bacterial physiology (Figure 2 and Figure 3) and colonization dynamics (Figure 4), we next sought to investigate the underlying metabolic mechanisms. To gain insight into these effects, we performed targeted metabolomic analyses comparing Δ*tktA* and Δ*talB* mutants to the wild-type strain (Figure 5). A Principal component analysis showed that the three strains form distinct clusters, indicating major differences in global metabolic profiles (Figure 5a). Both mutants exhibited extensive alterations in central metabolism, revealing distinct and non-overlapping metabolic signatures (Figure 5b).

The Δ*talB* mutant displayed relatively limited metabolic perturbations (Figure 5b,c), consistent with its preserved growth phenotype. Overall, central carbon metabolism remained largely intact, as most intermediates of glycolysis (e.g., glucose-6-phosphate, phosphoenolpyruvate) and the TCA cycle (e.g., citrate) were maintained at levels comparable to those of the wild-type strain. This indicates that, in contrast to TktA, TalB is not essential for sustaining carbon flux through the main energy-generating and biosynthetic pathways of central metabolism under the tested conditions. Nevertheless, specific alterations were detected in pathways connected to the PPP and nucleotide biosynthesis. Although NADPH levels were not critically reduced, a marked increase in oxidized glutathione (GSSG) pointed to a moderate redox imbalance, suggesting that TalB contributes to optimal redox homeostasis despite being dispensable for overall metabolic integrity. The Δ*talB* mutant also exhibited an accumulation of N-carbamoyl-aspartate, dihydroorotate, and orotate, suggesting a partial bottleneck in carbon flux toward pyrimidine biosynthesis. In addition, increased levels of glucose and aspartate were observed, indicative of metabolic rewiring and redistribution of carbon sources.

In contrast, the Δ*tktA* mutant exhibited profound metabolic alterations (Figure 5b,d), indicative of a global collapse of central carbon metabolism. A severe depletion of key metabolites was observed, notably NADPH, which was nearly undetectable, alongside a strong accumulation of oxidized glutathione, reflecting a major disruption of redox balance. Central metabolic pathways were broadly impaired, as illustrated by the marked reduction of TCA cycle intermediates such as succinate and oxaloacetate. Nucleotide biosynthesis was also affected, with a strong decrease in IMP, XMP and hypoxanthine. Concomitantly, several upstream intermediates accumulated in the Δ*tktA* mutant, including ribose phosphate, N-carbamoyl-aspartate, and dihydroorotate. This pattern is consistent with the presence of a metabolic bottleneck resulting from the inability to rearrange carbon skeletons in the absence of transketolase activity. While sedoheptulose-7-phosphate levels were reduced in the Δ*tktA* mutant, other key non-oxidative PPP intermediates, such as erythrose-4-phosphate and fructose-6-phosphate were not measured in the targeted metabolomic dataset, limiting a comprehensive assessment of carbon redistribution within this pathway. Altogether, these findings indicate that TktA is essential for maintaining carbon flux distribution, supporting biosynthetic pathways, and preserving redox homeostasis.

Taken together, these results highlight a clear functional hierarchy within the non-oxidative PPP. While TalB contributes to the fine-tuning of metabolic fluxes and redox balance, its loss can be compensated without major disruption of cellular metabolism. In contrast, TktA plays a central and non-redundant role, and its absence results in a systemic metabolic failure incompatible with normal bacterial physiology.

## 4. Discussion

Our results establish the non-oxidative PPP as a central determinant of *Y. pestis* adaptation to the flea environment, with TktA and TalB fulfilling distinct and non-redundant functions. Beyond its canonical role in biosynthesis, our data support a model in which metabolism contributes actively to infection-related processes, in which the organization and distribution of metabolic fluxes are likely to influence bacterial fitness, persistence, and transmission. By integrating metabolomic and infection data, we show that the non-oxidative PPP appears to be hierarchically organized, coupling carbon flux distribution to biofilm formation and sustained colonization.

Consistent with previous reports that disruption of PPP enzymes such as RpiA1/RpiA2 or Rpe abolishes flea blockage without preventing initial colonization [7], the Δ*talB* mutant retains early colonization capacity but fails to reach wild-type bacterial load over time in the proventriculus (Figure 4). However, the phenotypes associated with these enzymes are not identical, suggesting a functional specialization within the non-oxidative PPP. RpiA1/RpiA2 and Rpe catalyze reactions directly linked to ribose-5-phosphate and pentose interconversion, processes that support early resistance to the detrimental flea gut environment and, after bacterial adaptation, contribute to proventricular biofilm formation [7]. In contrast, TalB appears to play a more modulatory role, contributing primarily to the maintenance of metabolic homeostasis within the proventriculus rather than to the initial establishment of colonization. TktA occupies an even more central position, as its disruption results in severe metabolic collapse and growth impairment. Together, these observations support a hierarchical organization of the non-oxidative PPP in *Y. pestis*, in which different enzymes contribute unequally to metabolic adaptation, biofilm formation, and flea colonization dynamics. There are several lines of evidence suggesting that *Y. pestis* relies on the flea gut for amino acids rather than hexoses [14]. In this context, the PPP may function as a metabolic integrator that redistributes carbon skeletons into biosynthetic pathways. The accumulation of pyrimidine intermediates in the Δ*talB* mutant is consistent with a defect in nucleotide flux balance (Figure 5).

Our findings are also consistent with studies performed in other pathogens, which collectively support the idea that the PPP functions as a central metabolic hub coordinating adaptation to host-associated environments. In *Francisella novicida*, disruption of non-oxidative PPP enzymes such as *tktA*, *rpiA*, and *rpe* severely impairs intracellular multiplication and broadly alters central carbon metabolism, highlighting the importance of PPP-dependent metabolic connectivity during host adaptation [22]. Similarly, metabolomic analyses in *Staphylococcus aureus* demonstrated that PPP dysfunction affects nucleotide biosynthesis, oxidative stress resistance, and biofilm formation through altered carbon allocation and reduced pyrimidine metabolism [23]. These observations parallel the accumulation of pyrimidine intermediates and the biofilm defect observed in the Δ*talB* mutant in our study (Figure 3 and Figure 5). In *Toxoplasma gondii*, functional analyses further revealed that different PPP enzymes display markedly distinct contributions to growth, redox homeostasis, and virulence, emphasizing the metabolic flexibility and hierarchical organization of this pathway [24]. Together, these studies support the notion that individual PPP enzymes can fulfill non-equivalent physiological roles depending on the metabolic constraints imposed by the host environment. In the context of *Y. pestis*, the flea gut likely represents a nutritionally constrained and oxidative niche in which fine regulation of carbon redistribution, nucleotide homeostasis, and redox balance becomes particularly important for long-term persistence and biofilm-dependent transmission.

In contrast, TktA is essential for maintaining global metabolic integrity. The Δ*tktA* mutant exhibits a systemic collapse of central metabolism, including depletion of TCA intermediates, near-complete loss of NADPH, and severe redox imbalance (Figure 5). These defects are consistent with the role of transketolase as a key node linking carbon metabolism to biosynthetic and redox pathways. In the highly oxidative flea gut [25,26], the inability to sustain NADPH production would be expected to compromise antioxidant defenses, which may contribute to the growth defect observed in this mutant.

TalB, by contrast, may play a modulatory role. Although dispensable for growth, its loss results in reduced biofilm formation (Figure 3) and impaired persistence in the proventriculus (Figure 4). The associated metabolic phenotype suggests that TalB contributes to the fine-tuning of carbon flux distribution under fluctuating environmental conditions (Figure 5). The current metabolomic data do not allow us to determine whether these phenotypes result from a direct impairment of extracellular matrix biosynthesis or from indirect effects on metabolic adaptation. However, the accumulation of pyrimidine intermediates together with the moderate redox imbalance observed in the Δ*talB* mutant suggests that altered nucleotide homeostasis and oxidative stress adaptation may collectively contribute to the biofilm defect. In addition, the flea gut likely represents a nutritionally constrained environment in which subtle alterations in carbon redistribution may become particularly detrimental for long-term persistence. Similar links between PPP dysfunction, nucleotide metabolism, oxidative stress adaptation, and biofilm-associated phenotypes have been reported in other pathogens [22,23,24], supporting the idea that TalB contributes to metabolic robustness rather than acting solely through a single biosynthetic pathway. Given the energetic cost of extracellular polysaccharide production, this function may be important for sustaining biofilm-dependent blockage.

Together, these findings support a model in which the non-oxidative PPP operates as a regulatory metabolic node that links nutrient utilization, redox balance, and biosynthetic capacity to transmission. TktA ensures global metabolic connectivity, whereas TalB, and likely other PPP enzymes, optimizes flux distribution required to establish a transmissible infection. This hierarchical organization explains why disruption of PPP components selectively impairs transmission without uniformly affecting colonization.

More broadly, our work reinforces the concept that flea colonization represents a metabolically constrained environment in which infection outcome is not solely dictated by the availability of nutrients but is likely influenced by the ability of the bacterium to dynamically allocate metabolic fluxes in response to environmental constraints. Defining how metabolic fluxes are dynamically regulated in vivo will be critical to fully understand the metabolic logic underlying *Y. pestis* transmission. Our data support the idea that metabolic flux control mechanisms contribute importantly to the regulation of *Y. pestis* transmission dynamics, rather than being solely determined by the availability of nutrients. While nutrient availability is a prerequisite, the efficient allocation of metabolic resources—governed by enzymatic regulation and pathway dynamics—appears to act as the critical gating factor for successful transmission. The non-oxidative PPP thus emerges as a candidate hub linking metabolic state to ecological fitness in the flea vector.

## Figures and Tables

**Table 1:** Strains, vectors, and primers used in this study.

Strain, Plasmid or Primer	Description or Sequence (5′-3′) ^1^	Reference
*Y. pestis* strains		
KIM6+ (WT)	Wild-type strain pYV-negative strain	[18]
∆*tktA*	∆*tktA*::*aphA3′*, Kan^R^	[7]
*tktA* comp.	∆*tktA*::*aphA3′* p*tktA*	This study
∆*talB*	∆*talB*::*Sh ble*, Zeo^R^	[7]
*talB* comp.	∆talB::*Sh ble* p*talB*	This study
∆*hmsHFRS*	∆*hmsHFRS*::*aphA3′*, Kan^R^	[7]
Vectors		
p*tktA*	pCR-Blunt *tktA*, Kan^R^ Zeo^R^	This study
p*talB*	pCR-Blunt *talB*, Kan^R^ Zeo^R^	This study
pAcGFP	*Aequorea coerulescens* GFP, Amp^R^	Addgene
Primers		
*tktA*-F	CACCGCTATCCGCTCATCAT	This study
*tktA*-R	ACGGCTAATCGCTCTTTAGGG	This study
talB-F	TTGCTGCCTGCTTGCAATTC	This study
talB-R	TGCGCCACCTCATTCAGATA	This study

^1^ The *aphA3* and *Sh ble* genes confer resistance to kanamycin (KanR) and zeocin (ZeoR), respectively.

## Data Availability

All data are provided within the manuscript.

## Supplementary Materials

The following supporting information can be downloaded at: https://www.mdpi.com/article/10.3390/pathogens15060603/s1, Table S1: Raw data from the targeted metabolomic analysis.

## Abbreviations

The following abbreviations are used in this manuscript:

PPP	Pentose Phosphate Pathway
TCA	Tricarboxylic Acid Cycle
NADP	Nicotinamide adenine dinucleotide phosphate (oxidized form)
NADPH	Nicotinamide adenine dinucleotide phosphate (reduced form)
P	Phosphate
Kan	Kanamycin
Zeo	Zeocin
Amp	Ampicillin
GFP	Green fluorescent protein
BHI	Brain heart infusion
LB	Lysogeny broth
M9	M9 minimal medium
G6P	Glucose-6-phosphate
F6P	Fructose-6-phosphate
G3P	Glyceraldehyde-3-phosphate
PEP	Phosphoenolpyruvate
aKG	Alpha-ketoglutarate (2-oxoglutarate)
CoA	Coenzyme A
X5P	Xylulose-5-phosphate
E4P	Erythrose-4-phosphate
R5P	Ribose-5-phosphate
S7P	Sedoheptulose-7-phosphate
PC	Principal component
GSSG	Glutathione disulfide
